# Are Phthalate Exposure Related to Oxidative Stress in Children and Adolescents with Asthma? A Cumulative Risk Assessment Approach

**DOI:** 10.3390/antiox11071315

**Published:** 2022-07-01

**Authors:** Po-Chin Huang, Po-Keng Cheng, Hsin-Chang Chen, Ivy Shiue, Wan-Ting Chang, Hsin-I Huang, Jung-Wei Chang, I-Jen Wang

**Affiliations:** 1 National Institute of Environmental Health Sciences, National Health Research Institutes, Miaoli 35053, Taiwan; pchuang@nhri.edu.tw (P.-C.H.); ansd39@nhri.edu.tw (P.-K.C.); ivy.scthiue@gmail.com (I.S.); wtchang2@nhri.edu.tw (W.-T.C.); lotty430@nhri.edu.tw (H.-I.H.); 2 Research Center for Environmental Medicine, Kaohsiung Medical University, Kaohsiung 80756, Taiwan; 3 Department of Medical Research, China Medical University Hospital, China Medical University, Taichung 40402, Taiwan; 4 Department of Chemistry, Tunghai University, Taichung 40704, Taiwan; hsinchang@thu.edu.tw; 5 Institute of Environmental and Occupational Health Sciences, School of Medicine, National Yang Ming Chiao Tung University, Taipei 11221, Taiwan; 6 Department of Pediatrics, Taipei Hospital, Ministry of Health and Welfare, Taipei 11267, Taiwan; 7 College of Public Health, China Medical University, Taichung 40402, Taiwan

**Keywords:** children, asthma, phthalate exposure, oxidative stress, cumulative risk assessment

## Abstract

Childhood asthma has become one of the most common chronic diseases in children and adolescents. However, few case–control studies investigating the relationship between phthalate exposure and asthma in children and adolescents have been conducted, especially in Asia. Therefore, we assessed the potential associations between phthalate exposure and asthma among children and adolescents in Taiwan. Because various demographic and environmental variables may influence the incidence and prognosis of asthma, we performed a case–control study with propensity score matching. Out of 615 Childhood Environment and Allergic Diseases Study participants, we conditionally matched 41 children with clinically diagnosed asthma with 111 controls. We then analyzed 11 phthalate metabolites by using liquid chromatography with tandem mass spectrometry. Compared with the control group, the median urinary phthalate levels for most phthalate metabolites in the case group were slightly increased, including monomethyl phthalate, mono-n-butyl phthalate, monobenzyl phthalate, monoethylhexyl phthalate, mono-(2-ethyl-5-hydroxyhexyl) phthalate, mono-(2-ethyl-5-oxohexyl) phthalate, mono-(2-ethyl-5-carboxypentyl) phthalate, and mono-(2-carboxymethylhexyl) phthalate. Hence, our results suggest that phthalate exposure may be associated with the development of asthma. In addition, prenatal environmental factors, such as active or passive smoking during pregnancy, may increase the risk of asthma.

## 1. Introduction

Childhood asthma has become one of the most common chronic diseases in children and adolescents [1]. Asthma is a chronic lung disorder that involves inflammation and swelling of the airways, resulting in the production of a large amount of mucus. The muscles surrounding the airways also tend to contract, further restricting the already congested airways. Asthma presents with coughing, wheezing, and shortness of breath. Researchers have argued that a combination of genetic and environmental factors affecting the immune system is largely linked to the increased incidence of asthma [2,3,4,5].

Environmental chemicals, such as phthalates, phenols, and parabens, may impair a child’s immunological development and increase the risk of atopic illness and asthma [6,7]. Multiple animal studies have demonstrated links between reduced lung function, atopic dermatitis, and enhanced T helper 2 (T_H_2) cell response and phthalate exposure [6,8,9]. The activity of T_H_2 lymphocytes may influence the biological mechanisms of asthma and aeroallergies [10]. Moreover, prenatal phthalate exposure is associated with elevated oxidative stress biomarkers, poor birth outcomes, neurological and behavioral disorders, and an increased risk of asthma and atopic disease in children worldwide [11,12,13,14].

Multiple epidemiological studies have highlighted a link between phthalate exposure and asthma, finding that emissions from polyvinyl chloride materials act as the main source of phthalate exposure [15,16,17,18]. Since the 1970s, phthalate exposure has been associated with an increased prevalence of asthma, allergies, and related respiratory symptoms [19,20,21,22,23]. Since the late 1990s, multiple epidemiological studies have investigated the possible relationship between phthalate exposure and airway diseases in children. For example, some researchers have highlighted that the level of di(2-ethylhexyl) phthalate (*DEHP*) in indoor dust is significantly associated with the onset of asthma in children [24,25]. Other epidemiological studies have also linked phthalate exposure to the development of asthma and its symptoms [16]. Hsu et al. determined that increased exposure to monobenzyl phthalate (MBzP) increases the incidence of asthma [26]. In another study, Gascon et al. highlighted that prenatal exposure to DEHP and butyl benzyl phthalate (*BBzP*) increases the risk of asthma and respiratory tract infections during childhood [27]. Recent cross-sectional studies have discovered that children with high levels of urinary DEHP metabolites had an increased risk of asthma [28,29,30]. All of these epidemiological studies demonstrate the relationship between phthalate exposure and asthma. However, case–control studies investigating the relationship between phthalate exposure and asthma in children and adolescents have infrequently been conducted, especially in Asia.

In this study, we used a propensity score matching (PSM) approach to create a balanced covariate distribution between patient (case) and healthy (control) groups. We compared the levels of phthalate exposure among children and adolescents with or without asthma. We also assessed the potential relationship between phthalate exposure and asthma in a case–control setting.

## 2. Materials and Methods

### 2.1. Ethics Statement

The study protocol was approved by the Institutional Review Board of Taipei Hospital (TH-IRB-11-02). Written informed consent was obtained after the study, and the sampling process was explained to the parents of the children and to the adolescents and their parents.

### 2.2. Participants and Study Design

All children included in this study were part of the Childhood Environment and Allergic Diseases Study (CEAS) cohort in 2010 in Taipei, Taiwan [31]. Exclusion criteria included an inability to answer question in Chinese and congenital and chronic diseases. The anthropometric data of the children were measured and recorded according to a standard procedure. In addition, a standardized self-reported questionnaire including basic demographics, birth history, family income, parental history of allergic diseases, and history of environmental exposure to pets and tobacco smoke was used to interview the parents.

To assess the incidence of postnatal exposure to pets, carpets, and tobacco smoke, among other allergens, an experienced pediatric allergist administered the International Study of Asthma and Allergies in Childhood questionnaire. For the detailed interview process, please refer to our previous study [31]. According to the 2015 Global Initiative for Asthma guidelines, the pediatric allergist confirmed doctor-diagnosed asthma on the basis of the following three criteria: (i) in the absence of a cold, the recurrence of at least two of the three symptoms of asthma (i.e., coughing, wheezing, and shortness of breath) within the past 12 months; (ii) a doctor’s diagnosis of asthma with ongoing treatment; and (iii) a clinical response to treatment with β_2_-agonists or inhaled corticosteroids [32].

Here, we used a PSM approach, which, in clinical and epidemiological investigations, helps create a balanced covariate distribution between patient (case) and healthy (control) groups [33]. In this approach, standardized mean difference (SMD) is a commonly used and valid statistic for assessing the balance of covariate distribution between case and control groups. Another measure that allows for matched sample comparison is the variance ratio (VR). We thus set the proper matching criteria as follows: SMD < 0.1 and 0.5 < VR < 2. We also matched three control participants on the basis of their sex, age, body mass index (BMI), and date of enrollment within 3 months to one case after the completion of both the questionnaire and the urine specimens. Among 615 eligible participants, we excluded 115 participants without body weight or height measurement, 203 participants without urine samples, and 97 participants who only analyzed 4 phthalates metabolites. From the remaining 201 participants, we then employed PSM to select 41 children with clinically diagnosed asthma and 111 controls without (see Supplementary Table S1 and Figure S1).

In this study, Power is conventionally set at 0.80, which implies that a study investigating a true effect will correctly reject the null hypothesis 80% of the time and will report a false negative (commit a Type II error) in the remaining 20% of cases. The sample size estimate is ranged from 31 to 181, which means the number of our study participants achieved the requirement of the medium effect.

### 2.3. Analytical Method for Detecting Phthalate Metabolites

First, mid-stream urine in the morning was collected. In order to analyze phthalate metabolites, urine samples were collected in a polypropylene container and stored at −80 °C. Eleven phthalate metabolites, namely monoethylhexyl phthalate (MEHP), mono-(2-ethyl-5-oxohexyl) phthalate (MEOHP), mono-(2-ethyl-5-hydroxyhexyl) phthalate (MEHHP), mono-(2-ethyl-5-carboxypentyl) phthalate (MECPP), mono-(2-carboxymethylhexyl) phthalate (MCMHP), mono-n-butyl phthalate (MnBP), monoisobutyl phthalate (MiBP), monoethyl phthalate (MEP), monoisononyl phthalate (MiNP), MBzP, and monomethyl phthalate (MMP), were detected using liquid chromatography–electrospray ionization–tandem mass spectrometry (LC-ESI-MS/MS) [34]. Briefly, a urine sample (100 μL) was incubated at 37 °C for 90 min with ammonium acetate (20 μL, >98%; Sigma-Aldrich, St. Louis, MO, USA), β-glucuronidase (10 μL, *E. coli* K12; Roche Biomedical, Mannheim, Germany), and a mixture of 10 isotopic (^13^C_4_) phthalate metabolite standards (100 μL, Cambridge Isotope Laboratories, Andover, MA, USA). An online system was used together with LC-ESI-MS/MS (Agilent 1200/API 4000; Applied Biosystems, Foster City, CA, USA). The limits of detection (LODs) for MMP, MEP, MiBP, MnBP, MBzP, MEHP, MEHHP, MEOHP, MECPP, MCMHP, and MiNP were 0.3, 0.3, 1.0, 1.0, 0.3, 0.7, 0.3, 0.3, 0.3, 0.1, and 0.1 ng/mL, respectively. Each batch contained one blank repeated quality control (QC) sample and one spiked QC sample, with the concentration of the blank samples set below twice the detection limit. For the QC sample, a pooled urine sample was spiked with phthalate metabolite standards (20–50 ng/mL). The relative percentage difference between the repeated samples and the recovered spiked QC sample was within ±30%. Concentrations below the calibration curve were evaluated as 1/2 LOD value [35].

### 2.4. Analytical Method for Detecting Oxidative and Nitrosative Stress Biomarkers

We used isotope dilution LC-MS/MS to evaluate the levels of four oxidative and nitrosative stress biomarkers: 8-hydroxy-2′-deoxyguanosine (8-OHdG), 8-nitroguanine (8-NO_2_Gua), 8-isoprostaglandin F2α (8-isoPGF_2α_), and 4-hydroxy-2-nonenal-mercapturic acid (HNE-MA). We also assessed the levels of malondialdehyde (MDA) in urine samples by measuring the level of thiobarbituric acid–reactive substances. For more detailed information, please refer to Cheng et al. [36].

### 2.5. Estimating Daily Exposure to Phthalates

We estimated the daily intake (*DI*) of each phthalate from urinary phthalate metabolites. Equation (1) shows the formula used [37,38,39], where *UE* is the urinary excretion of the assessed urinary phthalate metabolites per gram of creatinine; *CE*_smoothed_ is the smoothed creatinine excretion rate, a value based on age, body weight (*BW*), height (*ht*), and sex, proposed by Mage et al. [40,41]; *F_UE_* is the molar fraction, which describes the molar ratio between the excreted amounts of specific metabolites of each phthalate corresponding to the dietary intake of the parent phthalate; *MW_d_* is the molar weight of the diester parent compound; *MW_m_* is the molar weight of the corresponding monoester.

(1) $$\mathrm{Daily}\;\mathrm{intake}\;\left(\mu g/kg/day\right)=\frac{UE\left(\mu g/g\right)\times CE_{\mathrm{smoothed}}\left(mg/day\right)}{F_{UE}\times BW\left(kg\right)\times 1000\left(mg/g\right)}\times \frac{MW_{d}}{MW_{m}}$$

$$\begin{array}{l} \mathrm{For}\;\mathrm{adults}\;(\geq 18\;\mathrm{years}\;\mathrm{old}):\\ CE_{\mathrm{smoothed}}=1.93\times \left(140-Age\right)\times BW^{1.5}\times ht^{0.5}\times 10^{-3}\ldots \left(\mathrm{male}\right)\\ CE_{\mathrm{smoothed}}=1.64\times \left(140-Age\right)\times BW^{1.5}\times ht^{0.5}\times 10^{-3}\ldots \left(\mathrm{female}\right)\\ \mathrm{For}\;\mathrm{minors}\;(\geq 3-<18\;\mathrm{years}\;\mathrm{old}):\\ CE_{\mathrm{smoothed}}=ht\times \left\{6.265+0.0564\times \left(ht-168\right)\right\}\ldots ht<168\;cm\ldots \left(\mathrm{male}\right)\\ CE_{\mathrm{smoothed}}=ht\times \left\{6.265+0\;2550\times \left(ht-168\right)\right\}\ldots ht\geq 168\;cm\ldots \left(\mathrm{male}\right)\\ CE_{\mathrm{smoothed}}=2.045\times ht\times \exp\left\{0.01552\times \left(ht-90\right)\right\}\ldots \left(\mathrm{female}\right) \end{array}$$

Equation (2) shows the formula used to assess the *DI* of *DEHP* and dibutyl phthalate (*DBP*), where *UE* is the urinary excretion of the assessed total urinary *DEHP* or *DBP* metabolites per gram of creatinine:

(2) $$\mathrm{Daily}\;\mathrm{intake}\;\left(\mu g/kg/day\right)=\frac{UE\left(\mathrm{moles}/g\right)\times CE_{\mathrm{smoothed}}\left(mg/day\right)\times MW_{d}}{F_{UE}\times BW\left(kg\right)\times 1000\left(mg/g\right)}$$

### 2.6. Tolerable Daily Intake and Reference Dose of Phthalates

The tolerable daily intake (*TDI*) values of seven phthalates, according to the European Food Safety Authority (EFSA) and the World Health Organization (WHO), are outlined in Supplementary Table S2 [39,42]. Although phthalates have been intensively studied for years, only a few studies have assessed the health risks of asthma. Therefore, in this study, we used the results of previous animal experiments on allergic or immune reactions for our reference doses (RfDs). We adopted the lowest-observed-adverse-effect levels (LOAELs) of Zou et al. for *DEHP* (3 mg/kg/body) [43], Li et al. for *DBP* (4 mg/kg/body) [44], and Sadakane et al. for diisononyl phthalate (*DiNP*; 15 mg/kg/body) [45]. We then used these LOAELs to evaluate no-observed-adverse-effect levels (NOAELs) according to the recommendations of the US Environmental Protection Agency. Generally, the *NOAEL* is equal to the *LOAEL* divided by a modifying factor (*MF*), which was 10 in this study. Similarly, the RfD is equal to the *NOAEL* divided by an uncertainty factor (*UF*), which was 100 in this study. This means that the RfDs of *DEHP*, *DBP*, and *DiNP* were 3, 4, and 15 μg/kg/day, respectively. The formulas for *NOAEL* and RfD are as follows.

(3) NOAEL=LOAEL/MF

(4) RfD=NOAEL/UF

### 2.7. Cumulative Risk Assessment: Hazard Quotient and Hazard Index

We used the hazard quotient (*HQ*) to calculate the risk posed by each phthalate to each participant. The formula for *HQ* is as follows:

(5) $$HQ=\frac{DI}{\mathrm{Reference}\;\mathrm{limit}\;\mathrm{value}}$$

A hazard index (*HI*) below 1 means a low probability of adverse effects from exposure to several chemicals [46]. The *HI* derived from *TDI* values is the sum of the HQs of *DEHP*, *DBP*, di-n-butyl phthalate (*DnBP*), diisobutyl phthalate (*DiBP*), *BBzP*, diethyl phthalate (*DEP*), and *DiNP* (*TDI* as the reference limit value). Similarly, the *HI* for asthma is the sum of the HQs of *DEHP*, *DBP*, and *DiNP* (RfD as the reference limit value).

(6) $$HI_{TDI}=HQ_{DEHP\;\mathrm{TDI}}+HQ_{DBP\;TDI}+HQ_{DnBP\;TDI}+HQ_{DiBP\;TDI}+HQ_{BBzP\;TDI}+HQ_{DEP\;TDI}+HQ_{DiNP\;TDI}$$

(7) $$HI_{asthma}=HQ_{DEHP\;RfD}+HQ_{DBP\;RfD}+HQ_{DiNP\;RfD}$$

### 2.8. Statistical Analysis

All descriptive statistics regarding participant demographics are presented as median and interquartile ranges for the continuous variables of age and BMI at enrollment and as percentages for the categories of family history of asthma, tobacco exposure, carpet exposure, pet exposure, and annual family income, among others. We calculated the detection rate as the percentage of samples with detectable phthalate metabolites among all samples. The distributions of urinary phthalate metabolites and oxidative and nitrosative stress biomarkers are described using detection rates, geometric means (GMs), and selected percentiles (25th, 50th, 75th, and 95th) for case and control groups. We used a Wilcoxon signed-rank test to compare the medians of urinary phthalate metabolites and oxidative and nitrosative stress biomarkers between the case and control groups. We also compared the concentrations of urinary phthalate metabolites across different demographic factors. Finally, we used logistic regression to obtain the odds ratios (ORs) of higher oxidative and nitrosative stress biomarkers with different HIs.

## 3. Results

### 3.1. Demographic Characteristics of the Study Participants

Table 1 shows the demographic characteristics of the study participants. No significant differences were observed in age, sex, or BMI. Mothers who smoked or were exposed to secondhand smoke during pregnancy were significantly more likely than their counterparts to have children with asthma (60.0% vs. 39.6%, *p* = 0.043). Moreover, low-income families were more likely than their counterparts to have children with asthma (59.4% vs. 24.8%, *p* = 0.001).

### 3.2. Distribution of Urinary Phthalate Metabolites and Oxidative and Nitrosative Stress Biomarkers

Table 2 compares the levels of phthalate metabolites between patients with asthma and healthy controls. Outliers, such as participants with phthalate metabolite levels exceeding 1000 ng/mL, were removed from the table. The detection rate of phthalate metabolites was similar in both the case and the control groups, ranging from 44.7% to 100%. In addition, MnBP (41.1), MECPP (36.5), and MEHP (36.1) exhibited the highest GMs of urinary phthalate metabolite concentrations (ng/mL) in the control group, whereas MECPP (53.7), MnBP (39.9), and MEHHP (34.9) exhibited the highest GMs in the case group. The median urinary phthalate levels in the case group were slightly higher than in the control group for most phthalate metabolites (MMP: 15.5 vs. 14.0; MnBP: 33.7 vs. 31.2; MBzP: 3.2 vs. 2.4; MEHP: 35.9 vs. 33.5; MEHHP: 50.0 vs. 28.93; MEOHP: 20.8 vs. 14.9; MECPP: 36.9 vs. 27.7; and MCMHP: 16.1 vs. 9.9 ng/mL). Table 3 shows the levels of oxidative and nitrosative stress biomarkers in the case and control groups.

### 3.3. Hazard Index and Distribution of the Daily Intake Values of Phthalates

**Figure 1:**
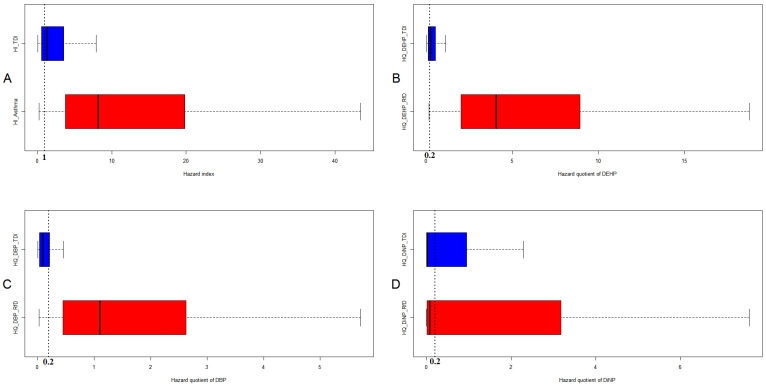
Comparisons of *HI* and HQs between RfD and TDI by boxplot. (**A**) *HI* (**B**) *HQ_DEHP_* (**C**) *HQ_DBP_* (**D**) *HQ_DiNP_*. A *HQ* of <0.2 is often considered acceptable, while a *HI* of <1.0 is considered acceptable.

Table 4 shows the estimated DI values (μg/kg/day) in the case and control groups. After comparing the DI value of each phthalate with the TDI and RfD values, we determined that the DI more commonly exceeded the RfD than the TDI. Furthermore, in the control group, *DEHP* exceeded the TDI in 16.8% of instances but exceeded the RfD in 85.3% of instances. Table 5 and Table 6 show the HQs and HIs by TDI and RfD for both the case and control groups. The HIs by TDI exceeding one were 68.4 and 65.8 for the control and case groups, respectively, whereas the HIs by RfD exceeding one were 94.7 and 97.4 for the control and case groups, respectively. Figure 1 compares the HIs and HQs between RfD and TDI.

### 3.4. Stratified Analysis of Urinary Phthalate Metabolites

Supplementary Table S3 shows the levels of urinary phthalate metabolites across different demographic factors in both the case and control groups. In the control group, participants with an annual income exceeding USD 31,250 had higher medians of urinary MMP levels (17.9 vs. 12.7 and 6.5 ng/mL) and urinary MBzP levels (3.8 vs. 2.4 ng/mL and ND) than those of their counterparts in other income categories. Participants who did not use incense at home had higher medians of urinary MMP levels (no: 23.9, yes: 8.8 ng/mL), urinary MiBP levels (no: 31.6, yes: 15.0 ng/mL), urinary MnBP levels (no: 97.6, yes: 24.3 ng/mL), and ΣDBPm (no: 0.8, yes: 0.17 nmol/mL) than those of their incense-using counterparts. Participants whose mothers did not smoke or were not exposed to secondhand smoke during pregnancy had a higher median urinary MEP level (no: 26.5, yes: 14.6 ng/mL) than that of their counterparts. Participants who did not have cockroaches at home had higher medians of urinary MEP levels (no: 43.5, yes: 17.9 ng/mL), urinary MiBP levels (no: 76.7, yes: 17.9 ng/mL), urinary MnBP levels (no: 89.6, yes: 25.6 ng/mL), ΣDBPm levels (no: 1.0, yes: 0.2 nmol/mL), and ΣDEHPm levels (no: 1.5, yes: 0.7 nmol/mL) than those of their counterparts. Participants who did not have pets at home had a higher median urinary MiNP level (no: 4.0 ng/mL, yes: ND) than that of their counterparts. Participants whose homes were located away 1 km from the main road had a higher median ΣDEHPm level (away: 2.2, within: 0.7 nmol/mL) than that of their counterparts. In the case group, participants who did not use incense at home had a higher median ΣDEHPm level (no: 1.4, yes: 0.5 nmol/mL) than that of their counterparts.

### 3.5. Association between Hazard Indices and Oxidative and Nitrosative Stress Biomarkers

As shown in Table 7, the adjusted ORs for higher oxidative and nitrosative stress biomarkers were stratified by *HI*. After adjustment for age, urinary creatinine, annual house income, and mother smoked or being exposed to secondhand smoking during pregnancy, *HI_TDI_* was significantly associated with higher 8-IsoPGF_2α_ as *HI_TDI_* ≥ 1.

## 4. Discussion

In this study, the median urinary phthalate metabolite levels in the case group were slightly higher than in the control group for most phthalate metabolites (i.e., MMP, MnBP, MBzP, MEHP, MEHHP, MEOHP, MECPP, and MCMHP). The levels of MMP, MEP, and MEHP were higher than those obtained by Hsu et al. [26], whereas the levels of MBzP, MEHHP, and MEOHP were lower. In addition, the levels of most urinary phthalate metabolites in this study were lower than those of Bertelsen et al. [28]. Hsu et al. reported that increasing the level of exposure to MBzP increases the incidence of asthma [26]. In another study, Bertelsen et al. reported that the presence of high levels of urinary *DEHP* metabolites in children increases the risk of asthma [28]. Wu et al. identified significant associations between the risk of asthma and the levels of MBzP (OR = 1.17, 95% confidence interval [CI]: 1.06–1.28), MEHHP (OR = 1.13, 95% CI: 1.03–1.24), and MECPP (OR = 1.20, 95% CI: 1.00–1.42) [47]. Overall, our results are consistent with previous studies, even at lower doses.

In their study, Bekö et al. did not observe a clear positive association between phthalate metabolites in urine and disease-specific case status. However, they highlighted an association for certain phthalates between nondietary phthalate exposure occurring in an indoor environment and allergic sensitization among children with asthma, rhinoconjunctivitis, and atopic dermatitis [29]. Generally, the effect of phthalate on a certain health indicator varies depending on the mode of exposure. However, the total intake of phthalates is calculated using the levels of urinary phthalate metabolites; this provides no information regarding the contribution of different exposure modes [48,49]. In addition, in an indoor environment, inhalation and dermal absorption may be more relevant in terms of health effects than total exposure, including dietary exposure. In this study, the fact that urinary phthalate metabolite exposure was regarded as total phthalate exposure may have been the reason why differences in urinary phthalate metabolites between the case and control groups were not significant.

Here, we proposed RfDs for *DEHP*, *DBP*, and *DiNP* and an *HI* for *asthma*. An *HI* value exceeding one was taken as indicative of the cumulative health risk of *asthma*. However, the TDI values set by the EFSA and WHO mostly pertained to reproductive adverse health effects and were, therefore, not suitable for this study. Because of the small number of studies on the assessment of the risk of *asthma* from phthalates, we used the results of previous animal experiments on allergic or immune reactions for RfDs. In an animal experiment involving injection and oral and nasal instillation, Zou et al. highlighted that although *DEHP* exposure did not affect immune responses in the absence of sensitization, *DEHP* increased the production of reactive oxygen species and MDA at 3 mg/kg/body. [43]. In another animal experiment, Li et al. used skin sensitization and fluorescein isothiocyanate to compare sensitized and nonsensitized groups. They reported that, in the absence of sensitization, exposure to 40.0 mg/kg/body *DBP* did not cause considerable allergic or immune responses. However, in the presence of sensitization, 4.0 mg/kg/body *DBP* caused substantial histological and immunological changes [44]. In another study, Sadakane et al. investigated the effects of *DEHP* and *DiNP* on the exacerbation of atopic dermatitis through a neck skin injection. However, they observed no significant differences in *DiNP* exposure at the highest concentration of 15 mg/kg/body [45]. Therefore, in this study, we adopted the LOAELs of Zou et al. for *DEHP* (3 mg/kg/body) [43], Li et al. for *DBP* (4 mg/kg/body) [44], and Sadakane et al. for *DiNP* (15 mg/kg/body) [45]. The RfDs that we obtained for *DEHP*, *DBP*, and *DiNP* were lower than the TDI values set by the EFSA and WHO, which resulted in a high rate of *HI* exceedance (larger than 1). This high rate of *HI* exceedance for *asthma* is indicative of the high risk of *asthma* due to phthalate exposure.

Kimber and Dearman investigated whether phthalates themselves are causative agents [50]. Some phthalates may function as adjuvants for agents that cause allergic sensitization. Using murine models (i.e., BALB/c mice), researchers have examined the potential of phthalates to function as adjuvants for immunological responses. Allergic *asthma* is characterized by immune responses involving antigen-presenting dendritic cells, T_H_2 cells, mast cells, and eosinophils, as well as the production of specific IgE, all of which underpin the development of allergic sensitization. In different situations, several phthalates may have immunomodulatory effects, immunosuppressive effects, or adjuvant effects on T_H_2 responses [15,16,50].

In our study, maternal active or passive smoking during pregnancy was significantly higher in children with *asthma* than in healthy children. Several studies have indicated that maternal smoking can lead to the onset of *asthma* during early childhood and even exacerbate its symptoms [51,52]. In addition, low-income families did have more mothers who smoked or were exposed to secondhand smoke during pregnancy in our study, which could be the potential reason why low-income families were more likely than their counterparts to have children with *asthma*. We also demonstrated that other risk factors of *asthma*, including exposure to secondhand tobacco smoke [53], a family history of *asthma*, and exposure to furry pets [54], were slightly higher in children with *asthma* than in healthy children.

Here, the diagnosis of *asthma* was made by a well-trained physician, thereby eliminating the risk of misclassification. We also evaluated the inclusion of the control group with a variety of strategies, which allowed us to maintain a large sample size and simultaneously ensure successful matching. However, the sample size used in this study was smaller than those of other studies, and the results may not be representative on a global scale. In addition, this was a case–control study in which causality cannot be established. We were also unable to determine whether patients with *asthma* changed their behavior after receiving their diagnosis. Although the half-life of phthalates is short, previous studies have only employed a single morning urine sample [55,56,57]. We did not have measures of *asthma* control (i.e., lung function, fraction exhaled nitric oxide, symptoms, exacerbations), which is another limitation.

## 5. Conclusions

The results obtained in this study indicate that environmental factors such as exposure to phthalates may be associated with *asthma* and its development in children. The median urinary phthalate levels in the case group were slightly higher than in the control group for most phthalate metabolites, although we were unable to pinpoint the reason. Further mechanistic, prospective, large-scale, epidemiological studies are required to verify the risk mechanism of *asthma*.

## Figures and Tables

**Table 1:** Characteristics of subjects in control (*n* = 111) and case (*n* = 41) groups.

Characteristics	Items	All Participants	Control (*n* = 111)	Case (*n* = 41)	*p*-Values ^a^
**Continuous variables**		Median (Range)	Median (Range)	Median (Range)	
Age (years)		7 (3–18)	7 (3–18)	7 (3–17)	0.479
BMI (kg/m^2^)		16.1 (13.2–23.4)	16.1 (13.2–23.2)	16.0 (13.4–23.4)	0.579
**Categorical variables**		*n* (%)	*n* (%)	*n* (%)	
Sex					0.993
	Male	113 (74.3)	82 (73.9)	31 (75.6)	
	Female	39 (25.7)	29 (26.1)	10 (24.4)	
Annual House income (USD) ^b^					0.001 **
	<18,750	44 (33.1)	25 (24.8)	19 (59.4)	
	18,750–31,250	37 (27.8)	33 (32.7)	4 (12.5)	
	>31,250	52 (39.1)	43 (42.5)	9 (28.1)	
Parents (at least one) have asthma or allergy ^c^					0.418
	Yes	18 (12.3)	11 (10.5)	7 (17.1)	
	No	128 (87.7)	94 (89.5)	34 (82.9)	
Mother smoked or being exposed to secondhand smoke during pregnancy ^d^					0.043 *
	Yes	66 (45.2)	42 (39.6)	24 (60.0)	
	No	80 (54.8)	64 (60.4)	16 (40.0)	
Children exposed to secondhand smoke ^e^					0.720
	Yes	88 (59.9)	62 (58.5)	26 (63.4)	
	No	59 (40.1)	44 (41.5)	15 (36.6)	
Home within 1 km from the main road ^f^					0.866
	Yes	120 (86.3)	88 (87.1)	32 (84.2)	
	No	19 (13.7)	13 (12.9)	6 (15.8)	
Cockroaches exist at home ^g^					0.271
	Yes	118 (79.7)	89 (82.4)	29 (72.5)	
	No	30 (20.3)	19 (17.6)	11 (27.5)	
Have pets at home ^h^					0.810
	Yes	29 (19.5)	20 (18.5)	9 (22.0)	
	No	120 (80.5)	88 (81.5)	32 (78.0)	
Incense use at home ^i^					0.671
	Yes	73 (50.3)	55 (51.9)	18 (46.2)	
	No	72 (49.7)	51 (48.1)	21 (53.8)	
Pesticide use at home ^j^					0.342
	Yes	57 (39.6)	39 (36.8)	18 (47.4)	
	No	87 (60.4)	67 (63.2)	20 (52.6)	
Indoor carpet ^k^					0.981
	Yes	13 (8.8)	10 (9.3)	3 (7.5)	
	No	134 (91.2)	97 (90.7)	37 (92.5)	
Have an air conditioner at home ^l^					1.000
	Yes	146 (100)	106 (100)	40 (100)	
	No	0 (0)	0 (0)	0 (0)	
Have mold plaque (wall cancer or moss) on the walls or bathroom at home ^m^					0.602
	Yes	53 (36.3)	37 (34.6)	16 (41.0)	
	No	93 (63.7)	70 (65.4)	23 (59.0)	
Have children take vitamin C or E ^n^					0.692
	Yes	47 (35.9)	38 (37.3)	9 (31.0)	
	No	84 (64.1)	64 (62.7)	20 (69.0)	
Have children take cod liver oil ^o^					0.182
	Yes	16 (12.3)	10 (9.8)	6 (21.4)	
	No	114 (87.7)	92 (90.2)	22 (78.6)	
Have children take deep sea fish oil ^p^					1.000
	Yes	9 (6.9)	7 (6.9)	2 (6.9)	
	No	122 (93.1)	95 (93.1)	27 (93.1)	
Have children take vitamin D and calcium tablets ^q^					0.896
	Yes	52 (40.0)	40 (39.2)	12 (42.9)	
	No	78 (60.0)	62 (60.8)	16 (57.1)	
Have children take lactic acid bacteria ^r^					0.441
	Yes	75 (58.1)	61 (60.4)	14 (50.0)	
	No	54 (41.9)	40 (39.6)	14 (50.0)	
Have children take propolis ^s^					0.673
	Yes	9 (6.9)	6 (5.9)	3 (10.3)	
	No	122 (93.1)	96 (94.1)	26 (89.7)	
Have children take traditional Chinese medicine (American ginseng) ^t^					0.488
	Yes	32 (24.4)	23 (22.5)	9 (31.0)	
	No	99 (75.6)	79 (77.5)	20 (69.0)	

^a^ Wilcoxon signed rank test (continuous variables) and Chi-square test (categorical variables) was applied to compare the control group and case group. * *p* < 0.05, ** *p* < 0.01, *** *p* < 0.001; parameters showing statistical significance are highlighted in bold. ^b^ Ten and 9 missing data of annual house income in control and case group, respectively; currency exchange rate of USD to new Taiwan dollar is 1:32. ^c^ Six missing data of parents (at least one) have asthma or allergy in control group. ^d^ Five and 1 missing data of mother smoked or was exposed to secondhand smoke during pregnancy in control and case group, respectively. ^e^ Five missing data of children exposed to secondhand smoke in control group. ^f^ Ten and 3 missing data of home within 1 km from the main road in control and case group, respectively. ^g^ Three and 1 missing data of cockroaches exist at home in control and case group, respectively. ^h^ Three missing data of have pets at home in control group. ^i^ Five and 2 missing data of incense use at home in control and case group, respectively. ^j^ Five and 3 missing data of pesticide use at home in control and case group, respectively. ^k^ Four and 1 missing data of have home carpeted in control and case group, respectively. ^l^ Five and 1 missing data of have an air conditioner at home in control and case group, respectively. ^m^ Four and 2 missing data of have mold plaque (wall cancer or moss) on the walls or bathroom at home in control and case group, respectively. ^n^ Nine and 12 missing data of have children take vitamin C or E in control and case group, respectively. ^o^ Nine and 13 missing data of have children take cod liver oil in control and case group, respectively. ^p^ Nine and 12 missing data of have children take deep sea fish oil in control and case group, respectively. ^q^ Nine and 13 missing data of have children take vitamin D and calcium tablets in control and case group, respectively. ^r^ Ten and 13 missing data of have children take lactic acid bacteria in control and case group, respectively. ^s^ Nine and 12 missing data of have children take propolis in control and case group, respectively. ^t^ Nine and 12 missing data of have children take traditional Chinese medicine (American ginseng) in control and case group, respectively.

**Table 2:** Levels of urinary phthalate metabolites (ng/mL) for control and case groups.

	*n*	%>LOD ^a^	GM (95% CI)	Min	Selected Percentiles				Max	*p*-Value ^b^
Phthalate Metabolites					25th (95% CI)	50th (95% CI)	75th (95% CI)	95th (95% CI)		
MMP										1.000
Control	95	91.6	10.7 (7.6–15.1)	ND	5.9 (3.8–7.2)	14.0 (8.5–17.5)	29.7 (23.2–40.3)	105.1 (75.3–129.8)	365.9	
Case	38	92.1	10.8 (6.3–18.6)	ND	4.6 (2.8–9.9)	15.5 (5.5–24.9)	32.0 (23.7–36.1)	79.3 (36.1–159.6)	159.6	
MEP										0.712
Control	95	88.4	14.2 (9.2–21.9)	ND	7.6 (4.1–11.9)	18.7 (13.8–26.1)	54.2 (31.6–81.7)	254.5 (179.8–304.7)	449.2	
Case	38	92.1	13.5 (7.4–24.8)	ND	6.5 (2.4–11.6)	17.4 (10.1–31.3)	52.7 (27.3–75.4)	98.4 (75.4–246.2)	246.2	
MiBP										0.879
Control	95	96.8	22.3 (15.9–31.4)	ND	8.7 (5.9–11.0)	21.4 (16.1–26.1)	76.2 (32.2–102.8)	320.5 (258.6–491.0)	509.2	
Case	38	100	25.6 (16.8–39.0)	2.3	11.4 (6.2–16.2)	20.0 (15.5–30.3)	49.5 (25.9–152.4)	229.6 (152.4–307.3)	307.3	
MnBP										0.939
Control	95	97.9	41.1 (29.0–58.4)	ND	15.0 (9.1–19.5)	31.2 (21.8–88.7)	157.20 (100.6–243.0)	671.6 (327.1–844.3)	925.6	
Case	38	100	39.9 (24.3–65.8)	3.3	15.4 (5.2–23.6)	33.7 (21.7–90.5)	171.8 (38.0–230.8)	348.9 (230.8–811.9)	811.9	
MBzP										0.352
Control	95	56.8	1.3 (0.8–1.9)	ND	ND (ND–ND)	2.4 (ND–3.3)	5.7 (4.1–8.0)	19.8 (15.0–113.6)	477.8	
Case	38	57.9	1.6 (0.8–3.4)	ND	ND (ND–ND)	3.2 (ND–5.9)	11.1 (5.1–18.5)	24.9 (18.5–136.7)	136.7	
MEHP										0.624
Control	95	92.6	36.1 (23.9–54.5)	ND	19.5 (12.9–23.7)	33.5 (26.8–71.6)	143.9 (106.6–182.2)	411.2 (268.0–776.3)	931.3	
Case	38	94.7	33.8 (17.6–64.9)	ND	12.7 (5.3–24.5)	35.9 (16.9–79.8)	132.9 (48.3–272.0)	594.9 (272.0–908.2)	908.2	
MEHHP										0.442
Control	95	94.7	24.6 (17.1–35.5)	ND	9.6 (6.5–14.1)	28.9 (19.2–34.8)	84.2 (52.7–149.6)	240.1 (216.0–351.5)	444.5	
Case	38	100	34.9 (21.5–56.7)	2.2	10.7 (4.4–22.5)	50.0 (13.3–91.5)	111.5 (65.7–142.3)	285.7 (142.3–343.6)	343.6	
MEOHP										0.582
Control	95	85.3	11.0 (7.2–16.7)	ND	5.7 (2.6–7.9)	14.9 (10.3–21.0)	36.8 (31.1–86.0)	156.8 (121.4–200.0)	448.6	
Case	38	86.8	13.0 (6.5–26.1)	ND	6.3 (ND–12.4)	20.8 (7.7–36.6)	62.6 (31.0–86.2)	159.6 (86.2–289.3)	289.3	
MECPP										0.439
Control	95	94.7	36.5 (24.4–54.8)	ND	12.9 (7.8–18.6)	27.7 (22.4–95.0)	172.3 (120.8–279.8)	452.8 (380.9–605.6)	634.5	
Case	38	100	53.7 (32.6–88.7)	3.9	12.5 (9.3–27.6)	36.9 (23.9–153.5)	216.3 (125.6–289.2)	413.5 (289.2–774.9)	774.9	
MCMHP										0.585
Control	95	91.6	9.4 (6.5–13.5)	ND	4.4 (3.3–5.3)	9.9 (6.0–16.0)	26.8 (19.5–30.9)	182.5 (106.8–288.3)	333.8	
Case	38	89.5	9.5 (5.1–17.7)	ND	4.9 (2.5–9.3)	16.1 (7.3–23.7)	26.5 (20.0–30.5)	108.6 (30.50–342.4)	342.4	
MiNP										0.539
Control	95	48.4	1.7 (0.9–3.3)	ND	ND (ND–ND)	ND (ND–4.6)	17.1 (8.5–84.5)	343.2 (296.3–458.4)	526.7	
Case	38	44.7	1.1 (0.5–2.7)	ND	ND (ND–ND)	ND (ND–7.5)	15.8 (3.5–19.2)	109.5 (19.2–300.1)	300.1	
ΣDBPm (nmol/mL) ^c^										0.966
Control	95		0.3 (0.2–0.4)	<0.1	0.1 (0.1–0.2)	0.3 (0.2–0.6)	1.0 (0.8–1.6)	3.7 (3.0–5.2)	5.7	
Case	38		0.3 (0.2–0.5)	<0.1	0.1 (0.1–0.2)	0.3 (0.2–0.7)	1.2 (0.5–1.6)	2.3 (1.6–5.0)	5.0	
ΣDEHPm (nmol/mL) ^d^										0.970
Control	95		0.6 (0.5–0.8)	<0.1	0.2 (0.2–0.3)	0.8 (0.5–0.9)	1.7 (1.1–2.6)	3.9 (3.6–6.1)	8.0	
Case	38		0.6 (0.4–1.0)	<0.1	0.2 (0.1–0.5)	0.8 (0.3–1.2)	1.7 (1.1–2.8)	5.9 (2.8–8.0)	8.0	

Abbreviation: not detectable (ND), interquartile range (IQR), confidence interval (CI), mono-methyl phthalate (MMP), mono-ethyl phthalate (MEP), mono-n-butyl phthalate (MnBP), mono-iso-butyl phthalate (MiBP), monobenzyl phthalate (MBzP), mono-isononyl phthalate(MiNP), mono-ethylhexyl phthalate (MEHP), mono-(2-ethyl-5-oxo-hexyl) phthalate (MEOHP), mono-(2-ethyl-5-hydroxyhexyl) phthalate (MEHHP), mono-(2-ethyl-5-carboxypentyl) phthalate (MECPP), mono-(2-carboxymethylhexyl) phthalate (MCMHP). ^a^ Limit of detection, ND was calculated as half of detection limit. The limit of detection for MMP, MEP, MiBP, MnBP, MBzP, MEHP, MEHHP, MEOHP, MECPP, MCMHP, and MiNP were 0.3, 0.3, 1.0, 1.0, 0.3, 0.7, 0.3, 0.3, 0.3, 0.1, and 0.1 ng/mL, respectively. ^b^ Comparison of control and case groups by Wilcoxon signed rank test. * *p* < 0.05, ** *p* < 0.01, *** *p* < 0.001. ^c^ ΣDBPm = sum molar concentrations of MiBP and MnBP. ^d^ ΣDEHPm = sum molar concentrations of MEHP, MEHHP, MEOHP, MECPP and MCMHP.

**Table 3:** Levels of oxidative/nitrosative stress biomarker for control and case groups.

	*n*	%>LOD	GM (95% CI)	Min	Selected Percentiles				Max	*p*-Value ^a^
Phthalate Metabolites					25th (95% CI)	50th (95% CI)	75th (95% CI)	95th (95% CI)		
**MDA (μmol/L)**										0.708
Control	88	100	6.0 (5.2–6.9)	2.0	4.1 (3.0–4.2)	5.7 (4.8–6.8)	8.3 (7.2–10.6)	19.2 (13.8–30.3)	41.1	
Case	38	100	6.0 (4.8–7.5)	1.2	4.1 (2.6–4.9)	6.3 (4.2–7.7)	8.4 (7.7–12.8)	16.1 (12.8–29.9)	29.9	
**8–OHdG (μg/L)**										0.148
Control	88	100	4.2 (3.7–4.7)	0.9	2.9 (2.3–3.1)	4.1 (3.4–4.7)	6.4 (5.2–8.0)	10.5 (9.1–11.9)	13.0	
Case	38	100	3.6 (2.9–4.4)	0.9	2.4 (1.8–2.9)	3.4 (2.5–4.2)	5.2 (4.0–8.1)	10.0 (8.1–12.0)	12.0	
**8–NO_2_Gua (μg/L)**										0.649
Control	88	100	3.1 (2.7–3.6)	<LOQ	2.0 (1.4–2.4)	3.6 (2.7–4.3)	5.2 (4.6–6.0)	7.9 (6.7–8.6)	8.7	
Case	38	100	2.8 (2.2–3.6)	<LOQ	2.4 (1.1–2.8)	3.4 (2.6–3.8)	4.9 (3.8–6.0)	7.0 (6.0–8.0)	8.0	
**4–HNEMA (μg/L)**										0.412
Control	88	100	29.8 (26.4–33.6)	6.2	21.6 (17.9–23.6)	29.0 (24.1–36.9)	45.3 (37.8–53.0)	72.6 (64.0–79.8)	86.5	
Case	38	100	27.2 (21.9–33.8)	5.9	16.0 (13.2–20.4)	29.2 (18.1–34.8)	39.7 (32.8–66.2)	72.4 (66.2–80.1)	80.1	
**8–IsoPGF_2α_ (μg/L)**										0.871
Control	88	100	4.7 (4.1–5.2)	1.1	3.1 (2.5–3.8)	4.8 (4.0–5.8)	7.0 (6.3–8.4)	10.4 (9.6–13.5)	14.1	
Case	38	100	4.7 (3.7–5.9)	1.3	2.4 (1.9–4.4)	4.8 (3.9–5.9)	8.8 (5.6–11.2)	12.6 (11.2–12.8)	12.8	

LOQ: limit of quantitation, LOQ for 8-NO_2_Gua is 1 μg/L. ^a^ Comparison of control and case groups by Wilcoxon signed rank test. * *p* < 0.05, ** *p* < 0.01, *** *p* < 0.001.

**Table 4:** Levels of estimated daily phthalate intakes (μg/kg/day) for control and case groups.

	*n*	>TDI (%) ^a^	>RfD (%) ^b^	GM (95% CI)	Min	Selected Percentiles				Max	*p*-Value ^c^
						25th (95% CI)	50th (95% CI)	75th (95% CI)	95th (95% CI)		
*DEHP*											0.599
Control	95	16.8	85.3	13.03 (9.97–17.01)	0.51	5.80 (3.59–8.54)	13.06 (10.16–16.87)	30.30 (22.47–48.02)	99.64 (83.77–154.02)	306.23	
Case	38	7.9	89.5	11.84 (8.33–16.82)	1.63	6.05 (3.68–8.55)	9.89 (7.76–20.72)	23.68 (15.32–29.75)	62.31 (29.75–154.24)	154.24	
*DBP* (i+n)											0.700
Control	95	5.3	53.7	4.65 (3.48–6.22)	0.09	1.66 (1.20–2.64)	4.86 (3.01–5.82)	11.17 (8.53–16.11)	49.08 (33.09–60.90)	334.93	
Case	38	2.6	47.4	4.30 (3.10–5.96)	0.70	2.32 (1.54–2.79)	3.59 (2.56–5.38)	7.03 (4.88–12.58)	23.10 (12.58–67.57)	67.57	
*DnBP*											0.617
Control	95	16.8		2.20 (1.60–3.03)	0.02	0.77 (0.54–1.09)	2.35 (1.38–3.02)	5.64 (4.21–8.50)	23.15 (18.51–40.26)	204.60	
Case	38	5.3		1.97 (1.36–2.85)	0.18	0.91 (0.73–1.07)	1.48 (1.02–2.75)	4.38 (2.24–7.96)	10.77 (7.96–40.27)	40.27	
*DiBP*											0.856
Control	95	9.5		1.46 (1.07–1.98)	0.01	0.57 (0.42–0.75)	1.25 (0.99–2.18)	4.40 (2.46–5.60)	12.58 (9.21–32.47)	85.89	
Case	38	5.3		1.53 (1.11–2.11)	0.26	0.81 (0.48–1.20)	1.26 (1.02–1.61)	2.43 (1.60–4.94)	8.58 (4.94–18.56)	18.56	
*BBzP*											0.519
Control	95	0		0.07 (0.05–0.11)	<0.01	0.02 (0.01–0.03)	0.10 (0.04–0.16)	0.29 (0.19–0.37)	1.34 (0.76–3.55)	26.03	
Case	38	0		0.09 (0.05–0.16)	<0.01	0.04 (0.01–0.06)	0.11 (0.04–0.17)	0.30 (0.16–0.61)	1.01 (0.16–0.61)	2.85	
*DEP*											0.379
Control	95	0		0.85 (0.58–1.24)	0.01	0.37 (0.21–0.49)	0.92 (0.62–1.48)	3.49 (1.81–4.32)	8.89 (5.58–47.44)	56.05	
Case	38	0		0.74 (0.45–1.23)	0.02	0.31 (0.15–0.65)	0.93 (0.35–1.27)	1.60 (1.06–2.65)	6.84 (2.65–34.46)	34.46	
*DiNP*											0.472
Control	95	26.3	35.8	4.23 (2.18–8.22)	0.06	0.25 (0.15–0.37)	1.15 (0.62–11.26)	51.88 (24.31–141.90)	1789.25 (459.94–3519.32)	4065.69	
Case	38	15.8	31.6	2.48 (0.98–6.26)	0.06	0.18 (0.13–0.30)	1.22 (0.26–9.20)	22.84 (7.40–80.96)	251.28 (80.96–730.38)	730.38	
DMP											0.813
Control	95			0.60 (0.45–0.80)	0.02	0.27 (0.18–0.33)	0.67 (0.51–0.75)	1.49 (0.92–2.03)	6.37 (2.97–18.65)	22.48	
Case	38			0.56 (0.36–0.87)	0.01	0.34 (0.12–0.56)	0.71 (0.41–1.05)	1.34 (0.94–1.82)	2.67 (1.82–5.22)	5.22	

Abbreviation: confidence interval (CI), benzyl butyl phthalate (*BBzP*), di-butyl phthalate (*DBP*), di-iso-butyl phthalate (*DiBP*), di-n-butyl phthalate (*DnBP*), di-ethyl phthalate (*DEP*), di-2-ethylhexyl phthalate (*DEHP*), dimethyl phthalate (DMP), di-iso-nonyl phthalate (*DiNP*), tolerable daily intake (TDI), reference dose (RfD). ^a^ TDIs for *DEHP*, *DBP* (i+n), *DnBP*, *DiBP*, *BBzP*, *DEP*, and *DiNP* are equal to 50, 50, 10, 10, 50, 500, and 50 μg/kg/day, respectively. ^b^ RfDs for *DEHP*, DBP (i+n), and *DiNP* are equal to 3, 4, and 15 μg/kg/day, respectively. ^c^ Comparison of control and case groups by Wilcoxon signed rank test. * *p* < 0.05, ** *p* < 0.01, *** *p* < 0.001.

**Table 5:** Hazard quotients and hazard index by tolerable daily intake for control and case groups.

	*n*	>1 (%)	GM (95% CI)	Min	Selected Percentiles				Max	*p*-Value ^a^
					25th (95% CI)	50th (95% CI)	75th (95% CI)	95th (95% CI)		
*HQ_DEHP_*										0.599
Control	95	16.8	0.26 (0.20–0.34)	0.01	0.12 (0.07–0.17)	0.26 (0.20–0.34)	0.61 (0.45–0.96)	1.99 (1.68–3.08)	6.12	
Case	38	7.9	0.24 (0.17–0.34)	0.03	0.12 (0.07–0.17)	0.20 (0.16–0.41)	0.47 (0.31–0.60)	1.25 (0.60–3.08)	3.08	
*HQ_DBP_*										0.700
Control	95	5.3	0.09 (0.07–0.12)	<0.01	0.03 (0.02–0.05)	0.10 (0.06–0.12)	0.22 (0.17–0.32)	0.98 (0.66–1.22)	6.70	
Case	38	2.6	0.09 (0.06–0.12)	0.01	0.05 (0.03–0.06)	0.07 (0.05–0.11)	0.14 (0.10–0.25)	0.46 (0.25–1.35)	1.35	
*HQ_DnBP_*										0.617
Control	95	16.8	0.22 (0.16–0.30)	<0.01	0.08 (0.05–0.11)	0.23 (0.14–0.30)	0.56 (0.42–0.85)	2.31 (1.85–4.03)	20.46	
Case	38	5.3	0.20 (0.14–0.28)	0.02	0.09 (0.07–0.11)	0.15 (0.10–0.28)	0.44 (0.22–0.80)	1.08 (0.80–4.03)	4.03	
*HQ_DiBP_*										0.856
Control	95	9.5	0.15 (0.11–0.20)	<0.01	0.06 (0.04–0.07)	0.13 (0.10–0.22)	0.44 (0.25–0.56)	1.26 (0.92–3.25)	8.59	
Case	38	5.3	0.15 (0.11–0.21)	0.03	0.08 (0.05–0.12)	0.13 (0.10–0.16)	0.24 (0.16–0.49)	0.86 (0.49–1.86)	1.86	
*HQ_BBzP_*										0.519
Control	95	0	0.001 (0.001–0.002)	<0.001	<0.001 (<0.001–0.001)	0.002 (0.001–0.003)	0.006 (0.004–0.007)	0.027 (0.015–0.071)	0.521	
Case	38	0	0.002 (0.001–0.003)	<0.001	0.001 (<0.001–0.001)	0.002 (0.001–0.003)	0.006 (0.003–0.012)	0.020 (0.012–0.057)	0.057	
*HQ_DEP_*										0.379
Control	95	0	0.002 (0.001–0.002)	<0.001	0.001 (<0.001–0.001)	0.002 (0.001–0.003)	0.007 (0.004–0.009)	0.018 (0.011–0.095)	0.112	
Case	38	0	0.001 (0.001–0.002)	<0.001	0.001 (<0.001–0.001)	0.002 (0.001–0.003)	0.003 (0.002–0.005)	0.014 (0.005–0.069)	0.069	
*HQ_DiNP_*										0.472
Control	95	26.3	0.28 (0.15–0.55)	<0.01	0.02 (0.01–0.02)	0.08 (0.04–0.75)	3.46 (1.62–9.46)	119.28 (30.66–234.62)	271.05	
Case	38	15.8	0.17 (0.07–0.42)	<0.01	0.01 (0.01–0.02)	0.08 (0.02–0.61)	1.52 (0.49–5.40)	16.75 (5.40–48.69)	48.69	
*HI* (*HQ_DEHP_* + *HQ_DBP_* + *HQ_DnBP_* + *HQ_DiBP_* + *HQ_BBzP_* + *HQ_DEP_* + *HQ_DiNP_*)										0.356
Control	95	68.4	2.46 (1.68–3.60)	0.05	0.76 (0.44–1.02)	1.69 (1.33–2.42)	6.68 (3.95–12.20)	121.21 (39.25–259.17)	274.14	
Case	38	65.8	1.64 (1.04–2.58)	0.12	0.52 (0.38–1.14)	1.52 (0.94–2.09)	3.23 (2.00–7.97)	17.66 (7.97–52.13)	52.13	

Abbreviation: confidence interval (CI), hazard quotient (HQ), hazard index (*HI*). ^a^ Comparison of control and case groups by Wilcoxon signed rank test. * *p* < 0.05, ** *p* < 0.01, *** *p* < 0.001.

**Table 6:** Hazard quotients and hazard index by reference dose for control and case groups.

	*n*	>1 (%)	GM (95% CI)		Min	Selected Percentiles				Max	*p*-Value ^a^
						25th (95% CI)	50th (95% CI)	75th (95% CI)	95th (95% CI)		
*HQ_DEHP_*											0.599
Control	95	85.3	4.34 (3.32–5.67)		0.17	1.93 (1.20–2.85)	4.35 (3.39–5.62)	10.10 (7.49–16.01)	33.21 (27.92–51.34)	102.08	
Case	38	89.5	3.95 (2.78–5.61)		0.54	2.02 (1.23–2.85)	3.30 (2.59–6.91)	7.89 (5.11–9.92)	20.77 (9.92–51.41)	51.41	
*HQ_DBP_*											0.700
Control	95	53.7	1.16 (0.87–1.56)		0.02	0.42 (0.30–0.66)	1.21 (0.75–1.46)	2.79 (2.13–4.03)	12.27 (8.27–15.22)	83.73	
Case	38	47.4	1.08 (0.78–1.49)		0.17	0.58 (0.39–0.70)	0.90 (0.64–1.35)	1.76 (1.22–3.14)	5.77 (3.14–16.89)	16.89	
*HQ_DiNP_*											0.472
Control	95	35.8	0.28 (0.15–0.55)		<0.01	0.02 (0.01–0.02)	0.08 (0.04–0.75)	3.46 (1.62–9.46)	119.28 (30.66–234.62)	271.05	
Case	38	31.6	0.17 (0.07–0.42)		<0.01	0.01 (0.01–0.02)	0.08 (0.02–0.61)	1.52 (0.49–5.40)	16.75 (5.40–48.69)	48.69	
*HI* (*HQ_DEHP_* + *HQ_DBP_* + *HQ_DiNP_*)											0.320
Control	95	94.7	9.53 (7.12–12.77)		0.25	3.85 (2.63–4.68)	8.16 (5.81–10.84)	24.78 (13.01–37.90)	134.39 (48.75–322.41)	402.94	
Case	38	97.4	6.97 (4.87–9.96)		0.73	3.20 (1.62–4.85)	7.89 (4.21–10.29)	11.38 (9.82–19.84)	39.94 (19.84–73.70)	73.70	

Abbreviation: confidence interval (CI), hazard quotient (HQ), hazard index (*HI*). ^a^ Comparison of control and case groups by Wilcoxon signed rank test. * *p* < 0.05, ** *p* < 0.01, *** *p* < 0.001.

**Table 7:** Odds ratios ^a^ and 95% confidence intervals of higher ^b^ oxidative/nitrosative stress biomarkers with different hazard indices (N = 126).

		MDA		8-OHdG		8-NO_2_Gua		4-HNEMA		8-IsoPGF_2α_	
Hazard Index	Categories	ORs	95% CI	ORs	95% CI	ORs	95% CI	ORs	95% CI	ORs	95% CI
*HI_asthma_*	*HI* < 1 (Ref.)	1	-	1	-	1	-	1	-	1	-
	*HI* ≧ 1	0.58	0.02–8.80	1.90	0.16–44.35	2.43	0.22–54.68	2.41	0.22–54.11	3.06	0.27–71.28
*HI_TDI_*	*HI* < 1 (Ref.)	1	-	1	-	1	-	1	-	1	-
	*HI* ≧ 1	1.26	0.52–3.11	0.91	0.40–2.06	1.31	0.59–2.92	1.06	0.47–2.36	**3.83**	1.65–9.35

^a^ Adjusted for age, urinary creatinine, annual house income, and mother smoked or being exposed to secondhand smoke during pregnancy. ^b^ Oxidative/nitrosative stress biomarkers larger than 50th percentile of the population.

## Data Availability

The data presented in this study are available in this manuscript and supplementary material.

## Supplementary Materials

The following supporting information can be downloaded at: https://www.mdpi.com/article/10.3390/antiox11071315/s1, Table S1: Comparison of different case control ratio by propensity score matching; Table S2: Tolerable daily intake (TDI) of seven phthalates by EFSA and WHO; Table S3: Levels of urinary phthalate metabolites (ng/mL) for control and case groups at different demographic factors; Figure S1: Flow chart of the recruitment of the study.
