# Supplementary material for: Are Phthalate Exposure Related to Oxidative Stress in Children and Adolescents with Asthma? A Cumulative Risk Assessment Approach
Source: Antioxidants (Basel). 2022 Jul 1;11(7):1315. doi: 10.3390/antiox11071315 (PMC9312256; doi:10.3390/antiox11071315)
Supplement: Supplementary file 1 [file antioxidants-11-01315-s001.zip › antioxidants-1786747-supplementary.pdf]

Supplementary information

Missing data information in Table 1.

[b] Ten and 9 missing data of annual house income in control and case group, respectively.
[c] Six missing data of parents (at least one) have asthma or allergy in control group.
[d] Five and 1 missing data of mother smoked or was exposed to secondhand smoke during pregnancy in control and case group, respectively.
[e] Five missing data of children exposed to secondhand smoke in control group.
[f] Ten and 3 missing data of home within 1 km from the main road in control and case group, respectively.
[g] Three and 1 missing data of cockroaches exist at home in control and case group, respectively.
[h] Three missing data of have pets at home in control group.
[i] Five and 2 missing data of incense use at home in control and case group, respectively.
[j] Five and 3 missing data of pesticide use at home in control and case group, respectively.
[k] Four and 1 missing data of have home carpeted in control and case group, respectively.
[l] Five and 1 missing data of have an air conditioner at home in control and case group, respectively.
[m] Four and 2 missing data of have mold plaque (wall cancer or moss) on the walls or bathroom at home in control and case group, respectively.
[n] Nine and 12 missing data of have children take vitamin C or E in control and case group, respectively.
[o] Nine and 13 missing data of have children take cod liver oil in control and case group, respectively.
[p] Nine and 12 missing data of have children take deep sea fish oil in control and case group, respectively.
[q] Nine and 13 missing data of have children take vitamin D and calcium tablets in control and case group, respectively.
[r] Ten and 13 missing data of have children take lactic acid bacteria in control and case group, respectively.
[s] Nine and 12 missing data of have children take propolis in control and case group, respectively.
[t] Nine and 12 missing data of have children take traditional Chinese medicine (American ginseng) in control and case group, respectively.

Table S1. Comparison of different case-control ratio by propensity score matching

case-control ratio	1:3 (case= 46, control= 122) matching: sex, age, BMI		1:3 (case= 42, control= 117) matching: sex, age, BMI		1:3 (case= 46, control= 119) matching: sex, age		1:3 (case= 41, control= 111) matching: sex, age, BMI	
Variable	SMD	VR	SMD	VR	SMD	VR	SMD	VR
LPS	0.110	1.003	0.147	1.329	0.109	1.149	**0.080**	**1.271**
Age	-0.009	1.399	0.060	3.047	0.099	2.443	**0.052**	2.953
Sex	0.121	0.876	0.163	0.331	0.077	0.902	**-0.039**	**0.970**
BMI			0.022	0.973			**0.100**	**1.100**

SMD= Standardized mean difference; VR= Variance ratio; LPS= logit of the propensity score

Criteria of proper matching: SMD<0.1, 0.5<VR<2

Table S2. Tolerable daily intake (TDI) of seven phthalates by EFSA and WHO

**Parent phthalate Compounds**	**Endpoint**	**TDI (μg/kg /day)**
**DEP**	Organ weight	500
**DBP (i+n)**	Reproductive effect	50
**DnBP**	Reproductive effect	10
**DiBP**	Reproductive effect	10
**BBzP**	Reproductive effect	50
**DiNP**	Reproductive effect	50
**DEHP**	Reproductive effect	50

Table S3. Levels of urinary phthalate metabolites (ng/mL) for control and case groups at different demographic factors.

Control, median (interquartile rage)	**MMP**	**MEP**	**MiBP**	**MnBP**
Male	16.54 (5.43-35.56)	19.16 (6.16-55.75)	22.38 (8.67-90.36)	84.01 (12.81-225.12)
Female	10.77 (6.95-26.73)	18.57 (11.41-62.64)	16.66 (9.35-64.81)	22.33 (16.77-63.29)
Annual House income < 18,750	6.48 (2.82-23.24)	14.78 (4.32-43.51)	16.16 (4.74-76.66)	21.78 (8.35-97.55)
Annual House income 18,750-31,250	12.65 (6.95-33.02)	18.57 (8.98-58.63)	26.50 (8.57-102.79)	34.23 (15.40-149.71)
Annual House income > 31,250	17.90 (8.73-38.47)	26.10 (12.90-67.91)	22.40 (11.49-90.51)	39.32 (20.52-224.86)
Parents (at least one) have asthma or allergy, Yes	11.07 (6.17-58.18)	20.30 (6.75-75.91)	18.49 (12.38-32.46)	88.89 (21.49-181.07)
Parents (at least one) have asthma or allergy, No	14.68 (5.88-34.00)	19.16 (9.16-55.75)	22.09 (8.83-100.27)	30.93 (13.50-188.87)
Mother smoked or was exposed to secondhand smoke during pregnancy, Yes	14.64 (5.36-49.40)	14.62 (4.54-28.20)	23.34 (8.63-99.82)	30.94 (10.78-146.65)
Mother smoked or was exposed to secondhand smoke during pregnancy, No	14.89 (6.61-33.50)	26.48 (12.12-62.94)	19.00 (9.06-88.39)	31.26 (17.70-234.99)
Children exposed to secondhand smoke, Yes	14.00 (5.36-38.31)	17.64 (5.87-58.01)	22.32 (8.63-106.24)	30.94 (15.46-184.35)
Children exposed to secondhand smoke, No	15.51 (7.80-29.68)	24.66 (12.15-65.67)	20.34 (9.41-68.85)	28.09 (16.57-184.51)
Home within 1 km from the main road, Yes	11.71 (5.91-31.67)	20.10 (8.70-62.43)	21.29 (8.64-91.38)	30.93 (15.60-200.59)
Home within 1 km from the main road, No	23.15 (15.31-40.29)	17.90 (11.43-56.15)	33.29 (12.71-107.39)	63.29 (21.60-164.67)
Cockroaches exist at home, Yes	13.97 (5.87-29.79)	17.90 (6.34-50.18)	17.92 (8.57-64.81)	25.62 (11.86-146.32)
Cockroaches exist at home, No	29.39 (6.55-47.34)	43.51 (20.21-115.85)	76.66 (17.22-305.70)	89.55 (28.00-310.54)
Have pets at home, Yes	12.09 (7.02-23.36)	11.19 (5.78-23.91)	16.53 (8.55-24.12)	23.84 (16.43-51.71)
Have pets at home, No	16.09 (5.98-37.31)	24.01 (11.43-63.94)	22.61 (9.51-95.24)	50.40 (16.85-227.12)
Incense use at home, Yes	8.77 (4.84-23.19)	16.16 (7.11-48.42)	15.03 (8.69-33.37)	24.34 (14.97-94.61)
Incense use at home, No	23.91 (6.95-40.81)	26.10 (13.16-80.68)	31.63 (16.14-251.68)	97.55 (20.13-297.30)

Table S3. (continued)

Control, median (interquartile rage)	**MMP**	**MEP**	**MiBP**	**MnBP**
Pesticide use at home, Yes	16.25 (6.44-43.61)	18.57 (7.09-60.64)	22.82 (9.26-105.09)	30.61 (17.01-211.62)
Pesticide use at home, No	15.31 (6.08-32.36)	19.90 (9.38-48.42)	21.82 (9.05-82.48)	31.28 (15.53-181.95)
Have home carpeted, Yes	11.10 (2.56-26.61)	9.38 (1.11-28.78)	15.05 (6.34-22.04)	31.94 (5.98-118.61)
Have home carpeted, No	15.92 (6.65-36.41)	21.88 (11.41-62.36)	22.40 (9.50-102.79)	31.28 (17.72-224.34)
Have mold plaque (wall cancer or moss) on the walls or bathroom at home, Yes	11.67 (6.48-38.19)	19.44 (9.09-46.65)	19.51 (9.50-80.96)	37.51 (18.48-149.71)
Have mold plaque (wall cancer or moss) on the walls or bathroom at home, No	15.82 (6.04-34.45)	19.39 (8.25-61.43)	22.38 (9.22-92.28)	29.97 (15.46-216.43)
Have children take vitamin C or E, Yes	15.51 (6.53-34.75)	18.76 (11.57-33.24)	22.38 (11.14-99.82)	35.13 (17.40-180.61)
Have children take vitamin C or E, No	13.33 (5.98-32.15)	25.71 (6.28-66.15)	22.32 (9.06-89.68)	30.94 (15.38-193.83)
Have children take cod liver oil, Yes	15.76 (5.01-27.83)	20.02 (7.20-51.43)	13.20 (9.39-52.65)	23.69 (16.95-206.54)
Have children take cod liver oil, No	14.68 (6.12-35.45)	20.10 (9.29-59.56)	22.94 (9.33-95.24)	32.76 (16.50-180.81)
Have children take deep sea fish oil, Yes	23.15 (17.83-37.60)	40.01 (17.72-111.19)	131.72 (16.25-187.92)	146.32 (28.45-271.67)
Have children take deep sea fish oil, No	13.97 (5.90-30.50)	19.90 (9.03-57.20)	21.82 (8.95-88.48)	31.24 (14.97-174.34)
Have children take vitamin D and calcium tablets, Yes	16.71 (6.36-42.33)	19.67 (14.19-59.27)	22.61 (11.49-169.79)	59.08 (21.63-227.12)
Have children take vitamin D and calcium tablets, No	12.20 (5.98-26.36)	22.29 (5.87-58.01)	19.95 (8.10-80.57)	29.97 (13.50-163.27)
Have children take lactic acid bacteria, Yes	16.25 (7.64-38.74)	20.30 (11.91-62.36)	21.82 (10.11-88.30)	39.32 (19.28-225.38)
Have children take lactic acid bacteria, No	10.64 (5.60-25.03)	20.38 (4.99-58.34)	22.94 (6.69-140.05)	29.97 (10.52-114.06)
Have children take propolis, Yes	13.79 (7.78-33.03)	32.91 (4.94-81.15)	28.09 (18.94-68.24)	91.59 (33.47-102.81)
Have children take propolis, No	14.68 (5.98-32.15)	20.10 (9.29-58.34)	22.09 (9.30-95.24)	31.26 (16.43-224.60)
Have children take traditional Chinese medicine (American ginseng), Yes	10.21 (5.61-50.46)	20.30 (9.83-50.61)	24.11 (8.52-95.54)	31.27 (14.97-228.85)
Have children take traditional Chinese medicine (American ginseng), No	15.72 (6.30-29.65)	19.90 (8.43-60.50)	22.37 (9.53-91.83)	31.28 (17.25-167.95)

Table S3. (continued)

Control, median (interquartile rage)	**MBzP**	**MiNP**	**ΣDBPm**	**ΣDEHPm**
Male	2.46 (ND-7.00)	1.17 (ND-55.28)	0.45 (0.10-1.59)	0.81 (0.26-2.63)
Female	2.60 (ND-4.45)	2.13 (ND-67.57)	0.17 (0.12-0.80)	0.84 (0.21-2.26)
Annual House income < 18,750	ND (ND-2.52)	6.76 (ND-299.04)	0.17 (0.06-0.93)	0.64 (0.14-1.33)
Annual House income 18,750-31,250	2.36 (ND-6.21)	2.23 (ND-17.10)	0.27 (0.10-1.60)	1.04 (0.25-2.94)
Annual House income > 31,250	3.83 (ND-8.27)	ND (ND-238.13)	0.43 (0.15-1.37)	0.89 (0.33-2.96)
Parents (at least one) have asthma or allergy, Yes	2.25 (ND-3.65)	ND (ND-152.07)	0.43 (0.17-0.92)	0.59 (0.32-3.43)
Parents (at least one) have asthma or allergy, No	2.46 (ND-7.00)	3.50 (ND-67.11)	0.25 (0.10-1.29)	0.83 (0.24-2.27)
Mother smoked or was exposed to secondhand smoke during pregnancy, Yes	2.31 (ND-6.58)	1.12 (ND-16.39)	0.27 (0.09-0.99)	0.97 (0.21-3.09)
Mother smoked or was exposed to secondhand smoke during pregnancy, No	3.36 (ND-7.81)	1.17 (ND-121.24)	0.25 (0.12-1.43)	0.79 (0.26-2.26)
Children exposed to secondhand smoke, Yes	2.36 (ND-6.75)	2.43 (ND-22.21)	0.27 (0.11-1.12)	0.93 (0.19-3.09)
Children exposed to secondhand smoke, No	3.54 (ND-7.81)	ND (ND-121.24)	0.33 (0.11-1.17)	0.79 (0.36-1.88)
Home within 1 km from the main road, Yes	2.41 (ND-7.12)	ND (ND-66.18)	0.25 (0.10-1.35)	0.71 (0.21-2.39)
Home within 1 km from the main road, No	3.26 (ND-8.79)	ND (ND-8.43)	0.43 (0.17-1.08)	2.24 (0.97-3.61)
Cockroaches exist at home, Yes	2.45 (ND-6.46)	3.02 (ND-84.45)	0.21 (0.09-0.98)	0.72 (0.21-2.26)
Cockroaches exist at home, No	3.83 (ND-9.37)	ND (ND-10.18)	0.96 (0.24-2.99)	1.51 (0.74-3.85)
Have pets at home, Yes	2.42 (ND-6.46)	ND (ND-3.12)	0.19 (0.13-0.60)	0.46 (0.19-1.93)
Have pets at home, No	2.61 (ND-7.12)	4.02 (ND-109.47)	0.45 (0.12-1.35)	0.88 (0.29-2.72)
Incense use at home, Yes	2.36 (ND-4.23)	ND (ND-14.11)	0.17 (0.10-0.73)	0.76 (0.26-1.48)
Incense use at home, No	4.08 (ND-8.29)	8.33 (ND-92.62)	0.80 (0.17-2.51)	1.09 (0.29-3.15)

Table S3. (continued)

Control, median (interquartile rage)	**MBzP**	**MiNP**	**ΣDBPm**	**ΣDEHPm**
Pesticide use at home, Yes	2.62 (ND-7.98)	ND (ND-14.98)	0.27 (0.13-1.24)	0.92 (0.27-2.88)
Pesticide use at home, No	2.60 (ND-6.15)	2.23 (ND-92.62)	0.27 (0.11-1.26)	0.80 (0.25-2.47)
Have home carpeted, Yes	1.26 (ND-3.59)	ND (ND-11.40)	0.23 (0.04-0.60)	0.59 (0.24-1.19)
Have home carpeted, No	2.97 (ND-7.33)	2.23 (ND-67.57)	0.27 (0.14-1.39)	0.87 (0.27-2.94)
Have mold plaque (wall cancer or moss) on the walls or bathroom at home, Yes	2.36 (ND-4.28)	ND (ND-23.98)	0.28 (0.16-1.08)	0.64 (0.27-1.28)
Have mold plaque (wall cancer or moss) on the walls or bathroom at home, No	2.99 (ND-8.67)	2.43 (ND-106.08)	0.26 (0.10-1.38)	1.04 (0.26-2.90)
Have children take vitamin C or E, Yes	2.72 (ND-6.02)	ND (ND-22.28)	0.37 (0.15-1.29)	1.01 (0.27-2.90)
Have children take vitamin C or E, No	2.58 (ND-7.97)	2.43 (ND-71.79)	0.27 (0.10-1.16)	0.81 (0.27-1.68)
Have children take cod liver oil, Yes	9.78 (3.10-22.73)	ND (ND-8.37)	0.17(0.11-1.16)	1.54 (0.57-2.78)
Have children take cod liver oil, No	2.56 (ND-6.56)	2.43 (ND-66.18)	0.28 (0.12-1.20)	0.86 (0.27-2.27)
Have children take deep sea fish oil, Yes	3.29 (2.80-5.36)	2.13 (ND-264.88)	1.64 (0.46-1.83)	1.24 (0.96-2.93)
Have children take deep sea fish oil, No	2.52 (ND-7.19)	ND (ND-44.85)	0.26 (0.10-1.10)	0.79 (0.26-2.48)
Have children take vitamin D and calcium tablets, Yes	2.99 (ND-7.88)	ND (ND-23.74)	0.49 (0.16-1.65)	1.05 (0.31-3.07)
Have children take vitamin D and calcium tablets, No	2.55 (ND-6.18)	2.43 (ND-79.77)	0.27 (0.09-0.95)	0.79 (0.26-1.95)
Have children take lactic acid bacteria, Yes	3.26 (ND-6.84)	2.23 (ND-65.72)	0.28 (0.14-1.34)	0.89 (0.32-2.69)
Have children take lactic acid bacteria, No	2.11 (ND-7.28)	ND (ND-86.79)	0.27 (0.09-1.09)	0.83 (0.21-2.10)
Have children take propolis, Yes	4.19 (1.10-16.28)	ND (ND-3.36)	0.63 (0.25-0.92)	1.90 (0.42-2.86)
Have children take propolis, No	2.55 (ND-6.89)	2.18 (ND-71.79)	0.27 (0.11-1.43)	0.83 (0.27-2.29)
Have children take traditional Chinese medicine (American ginseng), Yes	3.24 (ND-6.95)	ND (ND-14.79)	0.24 (0.11-1.52)	0.92 (0.31-2.81)
Have children take traditional Chinese medicine (American ginseng), No	2.37 (ND-7.10)	2.23 (ND-92.62)	0.27 (0.14-1.10)	0.84 (0.26-2.27)

Table S3. (continued)

Case, median (interquartile rage)	**MMP**	**MEP**	**MiBP**	**MnBP**
Male	15.70 (7.51-33.42)	21.35 (8.46-49.44)	20.41 (12.87-44.07)	32.65 (19.36-160.65)
Female	5.86 (3.91-20.07)	14.45 (7.43-37.71)	22.70 (10.37-195.40)	35.25 (11.10-345.21)
Annual House income < 18,750	20.28 (7.25-27.42)	16.00 (9.57-33.03)	20.41 (18.50-42.60)	34.81 (23.04-164.86)
Annual House income 18,750-31,250	8.76 (2.16-14.94)	27.91 (9.70-47.14)	15.58 (13.19-19.55)	14.61 (12.18-21.32)
Annual House income > 31,250	13.61 (5.54-24.91)	13.22 (6.05-33.14)	16.16 (8.64-101.22)	35.69 (10.32-90.50)
Parents (at least one) have asthma or allergy, Yes	14.96 (7.03-24.19)	10.10 (4.57-36.55)	18.84 (12.87-163.66)	25.70 (22.66-423.64)
Parents (at least one) have asthma or allergy, No	15.66 (4.74-31.95)	20.08 (9.07-52.71)	23.46 (10.70-85.67)	35.25 (15.44-176.72)
Mother smoked or was exposed to secondhand smoke during pregnancy, Yes	15.53 (5.00-24.68)	11.58 (3.88-56.93)	18.50 (8.52-65.17)	27.07 (9.10-180.47)
Mother smoked or was exposed to secondhand smoke during pregnancy, No	17.07 (4.50-34.06)	24.80 (13.04-39.52)	30.06 (18.17-110.95)	37.24 (24.89-141.35)
Children exposed to secondhand smoke, Yes	15.66 (4.72-28.17)	17.40 (4.57-58.85)	19.99 (9.31-99.80)	32.59 (11.57-183.77)
Children exposed to secondhand smoke, No	15.43 (9.11-31.38)	21.35 (11.03-35.76)	29.84 (17.40-32.36)	36.45 (22.07-130.46)
Home within 1 km from the main road, Yes	15.66 (5.44-29.94)	23.06 (8.84-62.64)	19.99 (11.43-97.22)	33.73 (15.64-155.77)
Home within 1 km from the main road, No	9.78 (4.59-18.33)	16.45 (5.36-34.12)	27.83 (17.05-184.16)	235.72 (49.66-383.89)
Cockroaches exist at home, Yes	14.96 (4.61-24.91)	16.00 (7.88-35.70)	19.98 (11.48-31.33)	30.36 (17.03-170.42)
Cockroaches exist at home, No	23.72 (3.98-36.55)	18.81 (7.92-64.31)	31.50 (10.84-217.95)	34.81 (10.88-235.89)
Have pets at home, Yes	15.43 (5.14-19.46)	31.33 (16.00-34.72)	19.98 (18.36-101.22)	131.67 (17.03-170.42)
Have pets at home, No	19.56 (4.60-33.19)	13.15 (5.81-56.93)	23.46 (11.43-65.17)	33.73 (15.18-185.44)
Incense use at home, Yes	14.52 (4.25-20.07)	20.08 (9.13-35.45)	19.41 (15.55-31.20)	25.99 (16.07-121.37)
Incense use at home, No	23.72 (9.89-28.93)	13.09 (9.04-71.11)	25.39 (11.48-101.10)	38.04 (23.63-205.28)

Table S3. (continued)

Case, median (interquartile rage)	**MMP**	**MEP**	**MiBP**	**MnBP**
Pesticide use at home, Yes	14.53 (4.84-19.13)	18.67 (8.80-32.69)	19.32 (14.58-48.76)	63.09 (16.07-176.72)
Pesticide use at home, No	23.57 (4.97-29.94)	26.77 (8.29-73.34)	23.72 (10.52-110.86)	31.51 (20.11-154.63)
Have home carpeted, Yes	4.18 (3.50-67.86)	34.72 (23.81-52.92)	19.98 (17.75-60.54)	25.70 (19.59-124.62)
Have home carpeted, No	15.62 (5.14-28.89)	16.00 (6.05-42.92)	20.41 (11.31-95.92)	34.81 (15.75-170.42)
Have mold plaque (wall cancer or moss) on the walls or bathroom at home, Yes	12.79 (4.06-25.00)	18.67 (8.05-62.64)	19.99 (15.60-97.22)	24.74 (15.18-75.14)
Have mold plaque (wall cancer or moss) on the walls or bathroom at home, No	15.43 (5.65-30.95)	13.22 (6.97-40.65)	20.41 (8.62-66.36)	36.45 (16.18-160.65)
Have children take vitamin C or E, Yes	13.61 (2.83-24.91)	16.00 (11.62-34.72)	20.00 (18.84-29.84)	30.36 (21.70-185.42)
Have children take vitamin C or E, No	17.58 (8.80-29.91)	22.87 (9.13-39.52)	25.90 (13.57-97.25)	36.07 (20.77-141.35)
Have children take cod liver oil, Yes	11.75 (5.61-21.60)	20.10 (11.94-28.01)	22.94 (16.64-28.86)	28.03 (16.53-75.46)
Have children take cod liver oil, No	19.87 (4.84-31.95)	20.08 (9.07-41.79)	25.90 (14.74-130.41)	37.24 (22.77-181.67)
Have children take deep sea fish oil, Yes	25.09 (24.68-25.50)	19.47 (15.54-23.39)	40.41 (33.16-47.66)	104.59 (67.48-141.71)
Have children take deep sea fish oil, No	14.96 (4.55-26.90)	21.35 (9.10-40.65)	20.41 (14.90-98.57)	35.69 (18.72-151.04)
Have children take vitamin D and calcium tablets, Yes	14.28 (5.28-25.16)	20.73 (3.88-45.63)	22.94 (10.88-106.98)	32.59 (20.57-100.70)
Have children take vitamin D and calcium tablets, No	19.87 (4.22-29.91)	20.08 (11.96-34.45)	25.90 (18.17-66.49)	38.21 (22.25-180.47)
Have children take lactic acid bacteria, Yes	17.21 (7.56-24.61)	33.93 (13.91-66.16)	27.87 (15.69-130.41)	63.09 (27.29-181.67)
Have children take lactic acid bacteria, No	19.05 (3.27-31.95)	12.99 (6.06-23.64)	20.97 (15.69-32.75)	30.12 (15.53-108.34)
Have children take propolis, Yes	3.06 (1.61-19.53)	85.96 (45.51-90.11)	18.84 (13.49-85.64)	21.70 (18.51-113.49)
Have children take propolis, No	17.58 (6.63-25.66)	20.08 (9.78-35.45)	23.72 (15.55-85.67)	36.07 (22.77-160.73)
Have children take traditional Chinese medicine (American ginseng), Yes	20.28 (4.18-25.91)	16.00 (12.89-34.72)	20.00 (18.84-31.33)	30.36 (25.70-38.04)
Have children take traditional Chinese medicine (American ginseng), No	15.33 (5.31-25.90)	24.33 (7.80-43.74)	25.68 (13.57-143.21)	37.42 (15.64-172.52)

Table S3. (continued)

Case, median (interquartile rage)	**MBzP**	**MiNP**	**ΣDBPm**	**ΣDEHPm**
Male	4.79 (ND-10.97)	ND (ND-15.88)	0.25 (0.16-1.14)	0.79 (0.24-2.08)
Female	2.04 (ND-14.03)	ND (ND-2.66)	0.76 (0.09-1.73)	0.82 (0.20-1.19)
Annual House income < 18,750	5.59 (ND-14.34)	ND (ND-15.83)	0.26 (0.19-0.99)	1.08 (0.39-1.80)
Annual House income 18,750-31,250	ND (ND-0.70)	ND (ND-25.94)	0.14 (0.11-0.18)	0.23 (0.15-0.40)
Annual House income > 31,250	2.04 (ND-8.71)	ND (ND-2.65)	0.25 (0.08-1.19)	0.78 (0.41-1.74)
Parents (at least one) have asthma or allergy, Yes	3.44 (ND-5.80)	ND (ND-17.60)	0.21 (0.17-2.85)	0.69 (0.35-1.36)
Parents (at least one) have asthma or allergy, No	4.51 (ND-12.01)	ND (ND-14.67)	0.27 (0.13-1.21)	0.85 (0.20-1.80)
Mother smoked or was exposed to secondhand smoke during pregnancy, Yes	3.15 (ND-7.87)	1.81 (ND-16.06)	0.23 (0.08-1.09)	0.80 (0.16-1.47)
Mother smoked or was exposed to secondhand smoke during pregnancy, No	6.20 (ND-16.82)	ND (ND-8.70)	0.27 (0.20-1.29)	0.78 (0.48-2.51)
Children exposed to secondhand smoke, Yes	3.69 (ND-8.31)	1.81 (ND-16.25)	0.42 (0.08-1.39)	1.00 (0.18-1.73)
Children exposed to secondhand smoke, No	5.59 (1.09-17.42)	ND (ND-5.32)	0.26 (0.17-0.88)	0.78 (0.46-2.35)
Home within 1 km from the main road, Yes	3.40 (ND-13.24)	ND (ND-16.06)	0.25 (0.14-1.09)	0.74 (0.19-1.76)
Home within 1 km from the main road, No	4.52 (0.97-9.38)	ND (ND-5.00)	1.28 (0.34-2.42)	1.14 (0.86-1.44)
Cockroaches exist at home, Yes	2.85 (ND-7.65)	ND (ND-15.69)	0.25 (0.16-1.05)	0.78 (0.28-1.45)
Cockroaches exist at home, No	10.64 (ND-21.60)	ND (ND-11.64)	0.29 (0.10-2.04)	1.19 (0.13-2.58)
Have pets at home, Yes	8.71 (ND-16.44)	ND (ND-ND)	0.76 (0.16-1.22)	0.28 (0.16-1.19)
Have pets at home, No	3.69 (ND-9.06)	1.38 (ND-15.84)	0.25 (0.14-1.22)	0.85 (0.38-1.80)
Incense use at home, Yes	3.40 (ND-15.23)	ND (ND-10.09)	0.22 (0.15-0.88)	0.53 (0.18-1.05)
Incense use at home, No	4.79 (ND-8.71)	ND (ND-15.69)	0.31 (0.16-1.46)	1.43 (0.69-2.97)

Table S3. (continued)

Case, median (interquartile rage)	**MBzP**	**MiNP**	**ΣDBPm**	**ΣDEHPm**
Pesticide use at home, Yes	3.15 (ND-8.02)	1.38 (ND-10.09)	0.60 (0.15-1.15)	0.78 (0.20-1.49)
Pesticide use at home, No	4.94 (ND-13.81)	ND (ND-15.72)	0.25 (0.14-1.47)	0.83 (0.36-1.94)
Have home carpeted, Yes	ND (ND-68.43)	ND (ND-8.04)	0.21 (0.17-0.83)	0.20 (0.19-3.53)
Have home carpeted, No	3.94 (ND-10.64)	ND (ND-15.69)	0.26 (0.14-1.19)	0.79 (0.28-1.74)
Have mold plaque (wall cancer or moss) on the walls or bathroom at home, Yes	2.04 (ND-7.23)	1.81 (ND-16.59)	0.22 (0.14-0.99)	0.46 (0.19-1.52)
Have mold plaque (wall cancer or moss) on the walls or bathroom at home, No	4.79 (ND-14.24)	ND (ND-11.84)	0.29 (0.13-1.36)	1.08 (0.43-1.63)
Have children take vitamin C or E, Yes	5.92 (ND-16.23)	ND (ND-15.69)	0.25 (0.18-0.92)	0.50 (0.20-1.43)
Have children take vitamin C or E, No	3.69 (ND-9.36)	ND (ND-8.70)	0.28 (0.15-1.20)	0.99 (0.38-1.94)
Have children take cod liver oil, Yes	ND (ND-5.78)	ND (ND-2.02)	0.23 (0.15-0.47)	0.56 (0.18-1.53)
Have children take cod liver oil, No	5.34 (0.62-12.01)	ND (ND-10.09)	0.45 (0.19-1.28)	0.99 (0.43-2.22)
Have children take deep sea fish oil, Yes	3.90 (2.03-5.78)	7.90 (4.00-11.79)	0.65 (0.45-0.85)	1.36 (1.14-1.58)
Have children take deep sea fish oil, No	3.94 (ND-11.77)	ND (ND-9.39)	0.26 (0.15-1.21)	0.78 (0.24-1.77)
Have children take vitamin D and calcium tablets, Yes	3.69 (ND-9.79)	ND (ND-9.92)	0.40 (0.14-0.79)	0.85 (0.19-1.57)
Have children take vitamin D and calcium tablets, No	5.34 (ND-11.54)	ND (ND-2.78)	0.28 (0.20-1.24)	0.99 (0.37-2.51)
Have children take lactic acid bacteria, Yes	6.78 (2.39-16.39)	ND (ND-2.02)	0.62 (0.22-1.21)	1.05 (0.35-1.69)
Have children take lactic acid bacteria, No	1.25 (ND-7.80)	ND (ND-11.42)	0.24 (0.14-0.87)	0.76 (0.23-2.22)
Have children take propolis, Yes	ND (ND-3.03)	ND (ND-150.11)	0.18 (0.14-0.90)	0.50 (0.33-0.96)
Have children take propolis, No	4.51 (ND-12.01)	ND (ND-10.09)	0.28 (0.17-1.15)	0.85 (0.28-1.80)
Have children take traditional Chinese medicine (American ginseng), Yes	2.35 (ND-11.30)	ND (ND-ND)	0.25 (0.18-0.59)	0.74 (0.28-0.92)
Have children take traditional Chinese medicine (American ginseng), No	4.51 (ND-9.60)	ND (ND-12.81)	0.40 (0.13-1.29)	1.31 (0.26-1.94)

Abbreviation: not detectable (ND).
Yellow color: comparison of different demographic factor by Wilcoxon signed-rank test or Kruskal-Wallis test and the groups' medians are significantly from each other, $p < 0.05$.

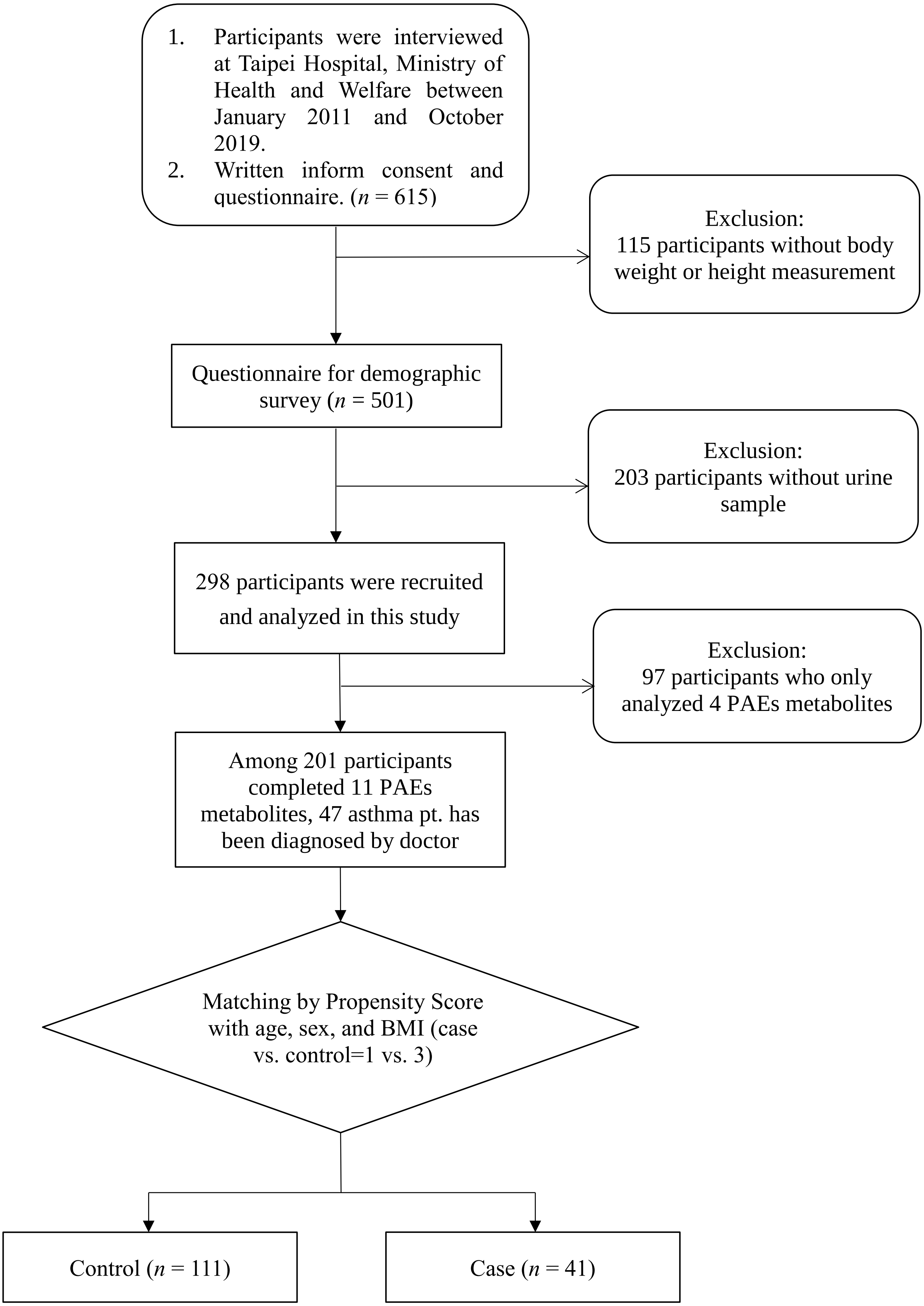

Figure S1. Flow chart of the recruitment of the study
